# Ubiquitin-specific protease 8 (USP8/UBPy): a prototypic multidomain deubiquitinating enzyme with pleiotropic functions

**DOI:** 10.1042/BST20190527

**Published:** 2019-12-17

**Authors:** Almut Dufner, Klaus-Peter Knobeloch

**Affiliations:** Institute of Neuropathology, Faculty of Medicine, University of Freiburg, Freiburg, Germany

**Keywords:** Cushings disease, DUBs, isopeptidase, ubiquitin proteasome system, UBPy, USP8

## Abstract

Protein modification by ubiquitin is one of the most versatile posttranslational regulations and counteracted by almost 100 deubiquitinating enzymes (DUBs). USP8 was originally identified as a growth regulated ubiquitin-specific protease and is like many other DUBs characterized by its multidomain architecture. Besides the catalytic domain, specific protein–protein interaction modules were characterized which contribute to USP8 substrate recruitment, regulation and targeting to distinct protein complexes. Studies in mice and humans impressively showed the physiological relevance and non-redundant function of USP8 within the context of the whole organism. USP8 knockout (KO) mice exhibit early embryonic lethality while induced deletion in adult animals rapidly causes lethal liver failure. Furthermore, T-cell specific ablation disturbs T-cell development and function resulting in fatal autoimmune inflammatory bowel disease. In human patients, somatic mutations in *USP8* were identified as the underlying cause of adrenocorticotropic hormone (ACTH) releasing pituitary adenomas causing Cushing's disease (CD). Here we provide an overview of the versatile molecular, cellular and pathology associated function and regulation of USP8 which appears to depend on specific protein binding partners, substrates and the cellular context.

## Introduction

The modification of proteins with the 76-amino-acid peptide ubiquitin represents perhaps the most versatile posttranslational modification system with innumerable layers of complexity and regulation [1]. The best-known functional consequence of protein ubiquitination is the targeting of substrates for degradation. However, virtually all cellular processes are regulated by ubiquitination. In addition to classical K48- and K63-linked polyubiquitination and monoubiquitination numerous other ubiquitin chain types are formed. This is possible as all seven lysines in ubiquitin and the N-terminal amine are accessible for linkage and chain formation. Furthermore, homotypic, heterotypic and branched chains can be formed and other ubiquitin-like modifiers, such as Nedd8 [2] or ISG15 [3] can be incorporated into ubiquitin chains. Moreover, residues other than lysines may be modified by ubiquitin [4]. SdeA, an effector protein of pathogenic Legionella pneumophila, was recently shown to mediate conjugation of phosphoribosylated ubiquitin to serine residues of protein substrates via a phosphodiesterbond [5]. It is not surprising that a plethora of ∼100 deubiquitinating enzymes (DUBs) is encoded in the human genome to counter-regulate this vast amount of modifications affecting most proteins in a cell. So far, seven DUB subfamilies have been identified including the UCH, OTU, MJD (Josephin), MINDY and ZUP1 cystein proteases, and the JAMM (MPN) metalloproteinases. The ubiquitin-specific proteases (USPs) form the largest subclass encompassing more than 50 cysteine proteases in humans [1,6]. Among these, USP8 represents a structurally unique [7], functionally promiscuous [8,9] and essential [6] DUB. The finding that mutations in *USP8* are associated with ACTH-secreting pituitary adenomas in CD has recently drawn much attention [10,11]. The underlying mechanism has been attributed to the most extensively studied canonical function of USP8 in protein trafficking and receptor tyrosine kinase (RTK) degradation [6]. In addition to highlighting findings on these major fields of USP8 research, we will discuss additional functions of USP8 that have emerged in recent years.

## The role of USP8 in endosomal sorting

**Figure 1.**
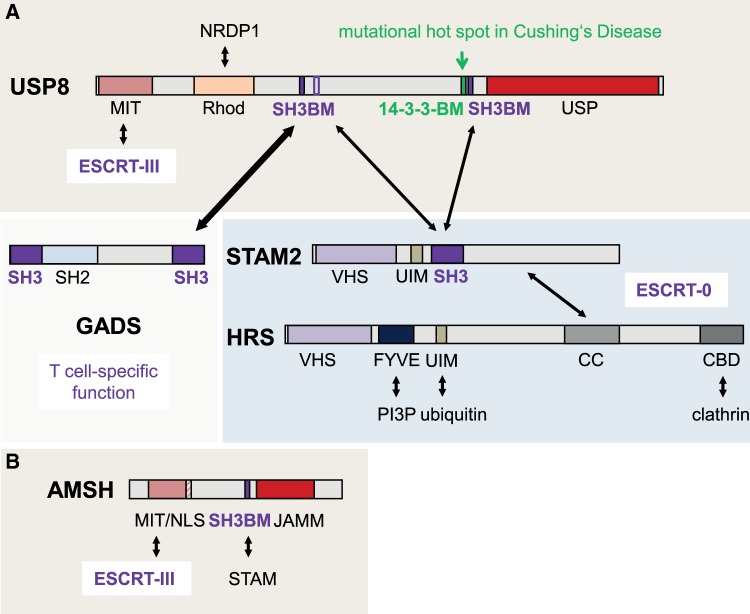
Domain structures and protein interaction partners of USP8 and AMSH. (**A**) Structure-function relationships of USP8 and interacting proteins and modules. The region affected by CD-causing mutations is depicted. (**B**) AMSH structure and interactions. ESCRT, Endosomal sorting complexes required for transport; FYVE, Zinc-binding domain that targets proteins to membrane lipids via interaction with phosphatidylinositol-3-phosphate, PI3P; VHS, Domain present in VPS-27, Hrs and STAM; MIT, microtubule interacting and transport; CBD, clathrin binding domain; JAMM, JAB1/MPN/Mov34 metalloenzyme; Rhod, rhodanese domain; UIM, ubiquitin interaction motif; CC, coiled-coil domain; NLS, nuclear localization signal.

USP8 contains an N-terminal microtubule interacting and transport (MIT) domain which has unveiled its potential to interact with CHMP proteins, components of the endosomal sorting complexes required for transport (ESCRT) III [12] (Figure 1A). ESCRT complexes mediate reverse topology membrane scission leading to the budding of vesicles ‘away from the cytosol’. This process is involved in multiple functions such as the generation of multivesicular bodies (MVBs) from endosomes or exosomal or viral budding [13–15]. USP8 also harbors at least two atypical central SH3-binding motifs (SH3BMs) [16,17] that flank a 14-3-3 protein binding motif (14-3-3BM). Remarkably, 14-3-3 protein interactions that depend on phosphorylation of the 14-3-3BM in USP8 were shown to inhibit USP8 activity *in vitro* and *in vivo* [18]. Mechanistically, 14-3-3 binding has been proposed to prevent the formation of a catalytically active USP8 cleavage product [10]. The SH3BMs were shown to mediate interaction with the SH3 domain present in signal-transducing adapter molecule 1/2 (STAM1/2) proteins, which together with Hepatocyte Growth Factor-Regulated Tyrosine Kinase Substrate (HRS) form the ESCRT-0 complex [16,17]. ESCRT-0 organizes ubiquitylated cargo such as receptor tyrosine kinases (RTKs) into flat clathrin-coated endosomal membrane areas prior to their interaction with ESCRT-I. ESCRT-0 does not directly participate in membrane budding and scission, but acts on the intermediate factors ESCRT-I, EXCRT-II and ALIX. Finally, ESCRT-III forms filaments involved in membrane remodeling and fission in a process controlled by the AAA ATPase VPS4. Although USP8 promotes epidermal growth factor receptor (EGFR) deubiquitination, its role in ESCRT-mediated endosomal sorting of RTKs remains controversial. While some studies favor a role in the promotion of EGFR degradation via trafficking to MVBs [19], others suggest a function of USP8 in redirecting the EGFR away from ESCRT-mediated degradation towards recycling [17,20,21]. Conflicting results could be caused by massive global ubiquitination and proteolytic stress triggered by depletion of USP8 or overexpression of a catalytically inactive enzyme. Furthermore, differential expression of regulatory RTK accessory proteins [22,23], or stabilizing posttranslational modifications of ubiquitin [24] may account for differential outcomes regarding the abundance of ESCRT cargo proteins in these studies. An additional layer in the regulation of cargo stability is based on the finding that USP8 ensures proper transport of lysosomal enzymes via retromer-dependent recycling of their receptor cation-independent mannose 6-phosphate receptor (ci-M6PR) to the trans-golgi network [25]. Remarkably, the metalloproteinase associated molecule with the SH3 domain of STAM (AMSH), which selectively cleaves K63-linked ubiquitin chains, also possesses an MIT domain and an SH3BM that interact with ESCRT-III components and STAM2, respectively (Figure 1B). AMSH may be more specifically involved in RTK recycling by outcompeting USP8 for binding to the ESCRT machinery [26,27]. The ESCRT-0 components HRS and STAM are massively destabilized in the absence of USP8 [12,19,21]. Of note, both HRS and USP8 were shown to be essential for cell viability [28]. In accordance with the finding that removal of ubiquitin from cargo proteins is required prior to their incorporation into internal MVB vesicles [29] more recent reports suggest that USP8 controls ESCRT-III function and the checkpoint responsible for transition of ubiquitinated cargo from ESCRT-0 to the final ESCRT-III complex, which does not bind ubiquitin. Ali et al. [30] propose that the ALIX-related ESCRT accessory protein HD-PTP/PTPN23 interacts with the EGFR, USP8 and the ESCRT-III subunit CHMP4B. In a sequence of competitive interactions, STAM2, which binds to HD-PTP/PTPN23 via two interactions, is replaced by CHMP4B and USP8 binding to both STAM2 and HD-PTP/PTPN23. Finally, STAM2 interaction with USP8 facilitates deubiquitination of the EGFR leading to its dissociation from ESCRT-0 and engagement with ESCRT-III. In yeast, Doa4 represents the likely orthologue of USP8 being involved in deubiquitination of intraluminal vesicle cargo. Analogous to the findings in the mammalian system, Doa4 restores supplies of unconjugated ubiquitin [31]. It is recruited by the ESCRT-III associated factor Bro1 and stabilizes ESCRT-III complexes, yet in a non-catalytic manner [29,32]. Using a cell-free system, Sirisaengtaksin et al. [33] confirmed that USP8 activity is critical for the single step of EGFR sorting into MVBs. Others suggest that USP8 counter-regulates EGF-induced ubiquitination of the ESCRT-III component CHMP1B, allowing it to assemble into a membrane-associated ESCRT-III polymer required for budding [34].

Besides the EGFR, the ubiquitination, endosomal lysosomal trafficking, and/or stability of many other transmembrane proteins have been shown to be regulated by USP8. These include the hepatocyte growth factor receptor MET [19], ERBB2 [35], the G protein-coupled receptor protease-activated receptor 2 [36]; chemokine receptor 4 [37], the Wg/Wnt receptor Frizzled [38],the calcium-activated potassium channel KCa3.1 [39], the epithelial Na+ channel ENaC [40], the low-density lipoprotein receptor (LDLR) [41,42], leucine-rich repeats and Ig-like domains 1 (LRIG1, a negative regulator of RTKs) [22,23], AMPA receptors [43], Tropomyosin related kinase A (TrkA) [44], vascular endothelial growth factor receptor 2 (VEGFR2) [45], β-site amyloid precursor protein-cleaving enzyme (BACE1) [46], connexin-43 [47], and leptin receptor [48]. Despite suggesting a common mode of action, conclusions on the impact of USP8 on protein stability in these studies are highly diverse and the precise molecular mechanisms remain elusive.

## *USP8* mutations cause Cushing's disease

**Figure 2.**
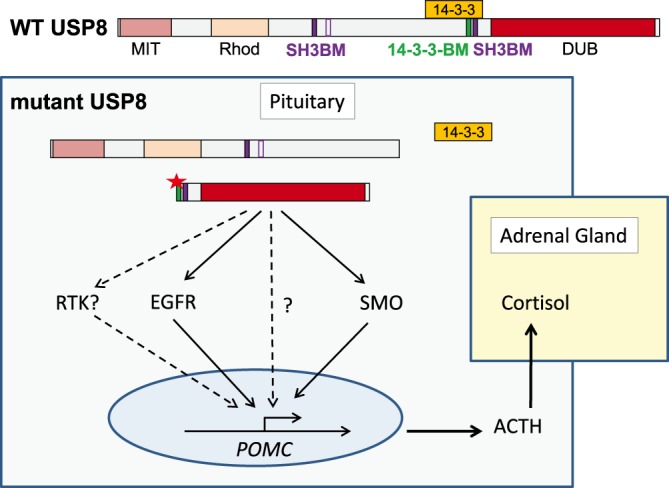
Mechanism of USP8-mediated cortisol hyperproduction in CD. 14-3-3 proteins fail to bind mutant USP8 leading to cleavage and constitutive activation of USP8. USP8-mediated stabilization and activation of the EGFR ultimately leads to increased transcription of the gene encoding the ACTH precursor proopiomelanocortin (*POMC*) and the development of corticotroph adenoma. Chronic elevation of ACTH is followed by excessive adrenal glucocorticoid secretion. The stabilization and activation of additional receptor tyrosine kinases, of SMO or deubiquitination of unknown nuclear targets of USP8 may also be involved in enhanced *POMC* transcription.

A recent key finding in USP8 research is the association of somatic mutations in the exon encoding the 14-3-3BM of USP8 with Cushing's disease (CD) [10,11]. CD is caused by ACTH-secreting pituitary adenomas leading to hypercortisolism associated with severe metabolic syndrome, infections, mood disorders, cerebral vascular disease and an increased cardiovascular risk. To date *USP8* mutations were found in ∼33% of all corticotropinomas [49]. The underlying mutations in *USP8* were shown to disrupt or diminish 14-3-3 protein binding (Figure 2). As a consequence, proteolytic cleavage of USP8 is enhanced leading to the generation of an activated catalytic fragment which due to diminished ubiquitination impairs the down-regulation of the EGFR [10]. Consequently, sustained EGFR signaling was identified as the cause of enhanced promoter activity of the gene encoding proopiomelanocortin (*POMC*), the precursor of ACTH. However, contrary to expectations, *USP8*-mutated pituitary adenomas displayed high immunoreactivity of USP8 in the nuclei, some of them exclusively, others at least partly [10,50]. Of note, not all USP8 variants associated with CD displayed higher deubiquitinating activity towards ubiquitinated EGFR than wild type USP8 [10]. Importantly, enhanced USP8 protease activity was closely linked to the occurrence of the 40kd C-terminal cleavage product harboring the catalytic domain [10]. USP8 activity also closely paralleled *POMC* promoter activation and ACTH production. Interestingly, we identified similar processing of USP8 in murine T cells upon activation of the T cell receptor (TCR), indicating that USP8 processing is not only a pathological process [51]. In contrast with Reincke et al. [10], Ma et al. [11] have investigated a larger cohort of corticotroph adenomas leading to the identification of 17 types of *USP8* mutations in the exon encoding the 14-3-3 binding region and of 3 prevalent mutations leading to the expression of USP8 variants, which proved to be most efficient in their ability to deubiquitinate the EGFR in the study of Reincke et al. [10]. The incidence of EGFR expression in *USP8*-mutated adenomas was 80% as compared with 50% in wild type USP8 expressing tumors [11]. However, *USP8* mutations were not associated with higher EGFR expression in other cohorts [50,52,53]. Hence, in view of the nuclear localization of USP8 variants found in CD, the variability of the effects of these mutations on USP8 activity towards ubiquitinated EGFR, and the rare occurrence of *USP8* mutations in other tumors, other USP8-dependent mechanisms than EGFR up-regulation cannot be ruled out to be responsible for CD pathogenesis (Figure 2). Preclinical studies have probed the sensitivity of primary CD tumor cells (including USP8 mutant cells), EGFR expressing AtT20 mouse corticotroph tumor cells and ACTH-secreting pituitary adenomas in transgenic mice with corticotroph-specific human EGFR expression to EGFR inhibitors like gefitinib demonstrating that these inhibitors are a treatment option for USP8 mutated corticotropinas [11,54,55].

Smoothened, a regulator of the Hedgehog pathway has also been shown to be a USP8 target substrate [56]. It is interesting to note that activation of the Hedgehog pathway induces ACTH secretion in a pathway which may be deregulated in *USP8* mutated corticotropinomas [57]. Recently, in USP8 wild-type corticotroph tumors somatic activating mutations were found to affect the catalytic domain of USP48 [58,59]. Substrates of USP48 include histone H2A and glioma-associated oncogene GLI1. The USP48 variant identified potentiated the stimulatory action of hypothalamic corticotropin-releasing hormone (CRH) on ACTH synthesis in a GLI1-dependent manner. Intriguingly, GLI1 is the downstream target of sonic hedgehog (SHH) signaling that is deregulated in corticotroph tumors [57] indicating that both USP8 and USP48 might trigger corticotroph tumorigenesis via the same pathway. *TP53* pathogenic variants were also identified in CD tumors, which similarly to increased H2A deubiquitination by USP48 may contribute to impaired DNA repair [58]. In view of the nuclear localization of mutant USP8 it remains to be determined whether USP8 also impinges more directly on tumor formation and *POMC* transcription in the nucleus.

Strikingly, one *de novo* germline heterozygous mutation was recently identified in the 14-3-3BM hotspot locus of the *USP8* gene [60]. The affected 16-year-old female patient displayed multiple medical problems including CD, developmental delay, ichthyosiform hyperkeratosis, chronic lung and kidney disease, hyperglycemia with a history of hyperinsulinemia, and partial growth hormone deficiency.

Loss of function mutations in *AMSH* lead to microcephaly-capillary malformation syndrome (MIC-CAP) characterized by small capillary malformations on the skin and severe microcephaly with associated symptoms [61]. Mechanistically these defects were linked to elevated RAS-MAPK and PI3K-AKT-mTOR signaling and ubiquitin-conjugated protein aggregate-induced progressive apoptosis, respectively. In contrast with *AMSH* knockout (KO) mice, which die between postnatal day 19 and 23 [62], *USP8* KO mice are embryonic lethal around E7.5 [21]. It is therefore not surprising that *USP8* germline loss of function mutations have not been reported in humans. These observations indicate that AMSH plays a more specific role in ESCRT-mediated processes which may be functionally complemented by the related AMSH-LP protein [63].

## USP8 in T cells

The N-terminal SH3BM in USP8 shows high affinity interaction with the SH3-domain containing adaptor molecule GRB2 related adaptor protein downstream of Shc (GADS) [51,64]. GADS is primarily involved in TCR signaling and USP8 is recruited to TCR-GADS enriched microclusters in a signaling status-dependent but GADS-independent manner. A pathway dependent on the central ESCRT-I component TSG101 has recently been shown to mediate exosome formation at the center of the immunological synapse which is largely devoid of TCR signaling [65]. Whether USP8 controls the segregation of exosomes for the transmission of transcellular signals across immunological synapses remains to be elucidated. Mice with a T cell-specific deletion of USP8 exhibit inflammatory bowel disease and dysfunctional regulatory T cells [51]. Employing this model system we showed that USP8 is critical for the positive selection of thymocytes without affecting TCR recycling. Adoro et al. [66] have suggested that CHMP5 is a critical target of USP8-mediated stabilization, which ensures thymocyte survival. They propose that CHMP5 stabilizes the anti-apoptotic Bcl2 protein via direct interaction in a manner independent of the ESCRT machinery.

## The function of USP8 in auto-/mitophagy and neurological disorders

In addition to its role in endosomal trafficking USP8 has been reported to be involved in mitochondrial quality control [67,68]. The E3-ubiquitin ligase Parkin represents a key player in the clearance of damaged mitochondria via autophagy (mitophagy) [68]. Both *Parkin* and the gene encoding the mitochondrial kinase PINK1 are mutated in familial Parkinson's disease (PD). During stress-induced mitophagy cytoplasmic Parkin translocates to dysfunctional mitochondria where it ubiquitinates a large number of substrates. Mitofusins, GTPases essential for mitochondrial fusion, are among the earliest targets undergoing proteasomal degradation. The recruitment of Parkin to damaged mitochondria is induced by PINK1 accumulating on damaged mitochondria and a feed-forward mechanism including mitofusin-2 phosphorylation [69], Parkin-autoubiquitination, substrate-ubiquitination, PINK1 autophosphorylation and PINK1-mediated phosphorylation of ubiquitin and the Parkin-ubl domain. Ubiquitination of mitochondrial proteins promotes the recruitment of ubiquitin-binding autophagy receptors such as sequestosome1 (SQSTM1)/p62 and NBR1. In an unbiased siRNA screen USP8 was identified as a DUB essential for the recruitment of Parkin to mitochondria in response to dissipation of the mitochondrial membrane potential [67]. USP8 was found to deubiquitinate Parkin specifically targeting K6 linked ubiquitin implicating that K6 linkages inhibit mitochondrial quality control when present at high levels on Parkin [67].

In addition, USP8 appears to be involved in insulin secretion via upstream regulation of Parkin-mediated mitophagy in pancreatic islet β-cells, which are particularly susceptible to mitochondrial dysfunction [70,71]. Pearson et al. [70] describe a regulatory complex consisting of the E3 ligases Clec16a and NRDP1, and USP8 which is critical for Parkin down-regulation by NRDP1 and fine-tuning of mitophagy. In this complex Clec16a stabilizes NRDP1 via non-degradable ubiquitination. Upon increased mitochondrial damage the complex is destabilized. Consequently, the function of USP8 in removing K6-linked ubiquitin from Parkin may become predominant leading to the mitochondrial translocation and activation of Parkin [71]. A DUB loss of function screen in Drosophila cells also revealed that USP8 stabilizes mitofusin. Consequently, genetic and pharmacological inhibition of USP8 normalized elevated mitofusin levels and prevented dopaminergic neuron loss in Drosophila PINK1 and Parkin KO flies [72].

Analysis of the role of USP8 in macroautophagy revealed that *USP8* loss of function in *Drosophila* leads to the accumulation of autophagosomes with non-degraded content due to a block of the autophagy flux. Unexpectedly, USP8 knock-down in HeLa cells resulted in deregulation of the autophagy flux [73]. This is consistent with recent findings suggesting that USP8 acts as a negative regulator of autophagy by deubiquitinating SQSTM1/p62 at K420 located in the UBA domain [74]. Interestingly, USP8 was also found to interact with NBR1 in a yeast two-hybrid screen [75]. Moreover, USP8 inactivation affected lysosomal biogenesis in *Drosophila* in a process which requires a functional endosomal pathway [73,76].

In Hela cells, USP8 was shown to ensure proper transport of lysosomal enzymes via retromer-dependent recycling of their receptor ci-M6PR to the trans-golgi network [25]. More recently, Gut et al. [77] performed a screen for molecules that promote autophagy in mouse embryonic stem cells (ESCs). ESCs rely on a high autophagic flux to allow for a fast metabolic rate and to maintain mitochondrial homeostasis. They found that EPG5, a regulator of autophagy promoting fusion of autophagosomes with lysosomes and/or late endosomes [78], is highly expressed in ESCs and critical for ESC pluripotency. They identified USP8 as an EPG5 interacting protein which regulates ESC self-renewal and pluripotency through removal of K63-linked ubiquitin from EPG5 at K252 leading to reinforcement of the interaction between EPG5 and LC3. Thus, USP8 appears to act on multiple levels to regulate proper execution of auto-/mitophagy in a cell type-specific manner.

A characteristic feature of PD is the formation of so-called Lewy bodies which represent ubiquitin-positive inclusions containing accumulated misfolded α-synuclein. Alexopoulou et al. [79] used patient samples and performed experiments in flies to provide evidence that USP8 stabilizes α-synuclein through deconjungation of K63-linked ubiquitin thereby increasing its toxicity. TDP-43 also forms characteristic insoluble protein aggregates found in multiple neurodegenerative diseases such as Alzheimer's disease (AD), frontotemporal lobar degeneration (FTLD) and amyotrophic lateral sclerosis (ALS). USP8 was identified as a TDP-43 interacting protein in a yeast-2-hybrid-screen and was shown to counteract TDP-43 ubiquitination [80]. In contrast with α-synuclein [79], USP8 deficiency enhanced TDP-43 neurotoxicity in *Drosophila*.

## USP8 controls hedgehog signaling

Members of the hedgehog (Hh/HH) family of secreted proteins function as morphogens governing embryogenesis, growth and patterning. Misregulation of HH signaling in vertebrates has been linked to many disorders including cancer [81,82]. Well-known components of the pathway include the transmembrane proteins Patched (Ptc/PTC) receptor and Smoothened (Smo/SMO), and the Cupidus interruptus (Ci)/GLI transcription factors in *Drosophila* and vertebrates, respectively. Upon binding of Hh/HH to Ptc/PTC, inhibition of Smo/SMO by Ptc/PTC is alleviated culminating in the activation of Ci/GLI proteins. The detailed process involving a plethora of additional regulatory and transport proteins is reviewed elsewhere [83]. While it has been clear that Hh/HH activates Smo/SMO by inducing Smo/SMO phosphorylation, only recently a parallel mode of regulation via modification with the Small Ubiquitin-like Modifier (SUMO) at K851 has been uncovered in *Drosophila* [84]. SUMOylation is triggered via dissociation of Smo from the de-sumoylating enzyme Ulp1 and was shown to allow recruitment of USP8 to antagonize Smo ubiquitination and degradation [56,84,85]. Thus, the regulation of Smo degradation exemplifies a pathway where ubiquitin- and SUMO-modification systems converge on the regulation of one common target.

## USP8 and the control of ciliogenesis

It has been shown that mammalian HH signaling depends on the presence of primary cilia to which SMO and other components of the pathway translocate to mediate activation of the GLI transcription factors [82,83]. Primary cilia are microtubule-based organelles which act as sensors involved in developmental signaling pathways [86]. The assembly of primary cilia is inhibited in dividing cells, but induced upon cell cycle exit signals. Recently, two groups have reported that USP8 participates in the control of ciliogenesis. However, their conclusions regarding its impact on ciliogenesis are contradictory [87,88]. Troilo et al. [87] identified USP8 as a hypoxia-inducible factor 1-α (HIF1α) deubiquitinating and stabilizing enzyme, which counteracts von Hippel-Lindau (VHL) tumor suppressor-mediated ubiquitination of HIF1α. They demonstrate that the maintenance of basal HIF1α expression in normoxia ensures the repression of the rab5 effector rabaptin5, a mechanism which is essential for endosome recycling-mediated ciliogenesis. Although loss of primary cilia is a key feature of VHL-deficiency, VHL does not affect ciliogenesis *per se* but rather secures primary cilium maintenance. Thus, VHL depletion from cells rescued their dependency on USP8 for cilia formation. In contrast, Kasahara et al. [88] reported that EGFR kinase suppresses ciliogenesis by phosphorylating USP8 on Tyr717 and Tyr810 enhancing the deubiquitinase activity. Consequently, the substrate trichoplein is stabilized by direct binding and deubiquitination. Trichoplein in turn binds and activates Aurora A kinase specifically at the G1 phase, which suppresses ciliogenesis. These results were validated in *USP8* KO zebrafish, which developed ciliopathy-related phenotypes. In addition, no reduction in HIF1α levels in USP8-depleted cells was detected. The data of Kasahara et al. [88] also point to a reciprocal relationship between primary cilia and cell proliferation which may provide further insights into mechanisms of tumorigenesis caused by dysregulated USP8.

## USP8 interactions with Nrdp1 and BRUCE

A common feature of DUB-regulated processes is the formation of regulatory complexes encompassing E3 ligases and DUBs exerting mutual regulation to fine-tune target modification as described for the regulation of Parkin by the E3 ligases Clec16a and NRDP1, and USP8 [70]. Initially, pulldown experiments with a C-terminal fragment of NRDP1 have revealed strong interactions not only with USP8, but also with the baculovirus IAP repeat (BIR)-containing ubiquitin-conjugating enzyme (BRUCE) [89,90]. While USP8 was found to stabilize Nrdp1 [90], Nrdp1 mediated ubiquitination and degradation of BRUCE [89]. Despite being a member of the inhibitor of apoptosis protein (IAP) family, BRUCE also has non-IAP functions such as the control of midbody ring formation during cytokinesis [91]. More recently, BRUCE was reported to act as a scaffolding protein during DNA double-strand break (DSB) repair forming a complex with USP8 and breast cancer susceptibility gene C terminus-repeat inhibitor of human telomerase repeat transcriptase expression 1 (BRIT1) [92,93]. As part of the complex, BRIT1 was proposed to be sequestered in a DSB-free chromatin region in unstimulated cells. Upon DSB induction, BRUCE promoted USP8-mediated deubiquitination of BRIT1 triggering its release and subsequent binding to γ-H2AX which is located in DSB-flanking chromatin where it facilitates chromatin relaxation. The promotion of BRIT1 function required the C-terminal Ubiquitin conjugating (UBC) domain of BRUCE in a mechanism that remains elusive [94]. Importantly, interactions of USP8 and BRUCE in the nucleus not only define a role of USP8 in the DNA damage response but may also point to a role in cytokinesis which would be in line with its profound role in regulation of the ESCRT machinery [95].

Nrdp1 also modulates the intracellular trafficking of three Jak-associated type I cytokine receptors, namely leptin receptor (LR), leukemia inhibitory factor receptor (LIFR), and interleukin-6 receptor (IL-6R) [96]. Mechanistically, the E3 ligase Nrdp1 indirectly destabilizes the ESCRT-0 complex by ubiquitinating and suppressing USP8. Consequently these receptors are rerouted from undergoing lysosomal degradation to compartments for ectodomain shedding leading to the enhanced release of soluble receptors by ADAM proteases.

## USP8 in Caspase8/cFLIP-controlled apoptosis

In line with its critical role in cell viability, USP8 was found to regulate apoptosis downstream of death receptors (DRs) [97,98]. In particular, it controls the FADD and procaspase-8 containing complexes which are formed upon stimulation of DRs. These include the death-inducing signaling complexes (DISCs) or internalized complexes called complexIIA and complexIIB depending on their precise composition and upstream DR [99]. Formation of these complexes culminates in the autoproteolytic cleavage and activation of procaspase-8, and the subsequent activation of effector caspases leading to apoptosis. However, the extent of caspase 8 activation is regulated by the presence of flice-like inhibitory protein (cFLIP) in the complex coming in two isoforms. cFLIP long (cFLIP_L_) is a procaspase-8-like protein lacking proteolytic activity. cFLIP short (cFLIP_S_) is a truncated version that lacks the caspase-like domain but is still able to form complexes with caspase-8. A current model suggests that the ratio of cFLIP to procaspase-8 determines the outcome of apoptosis, with cFLIP_S_ being a more stringent terminator of procaspase-8 activation than cFLIP_L_ [100].

One study showed that USP8 acts downstream of PTEN to enhance the ability of the E3 ligase Itch to reduce cFLIP_S_ stability and increase tumor necrosis factor-related apoptosis-inducing ligand (TRAIL) sensitivity in human glioblastoma multiforme cells [97]. However, Jeong et al. [98] showed that USP8 directly interacts with the caspase-like domain in c-FLIP_L_ to induce deubiquitination and stabilization of cFLIP_L_, but not cFLIP_S_. Depletion of USP8 destabilized cFLIP_L_ resulting in sensitization to DR-induced apoptosis. Moreover, USP8 depletion attenuated tumor growth upon TRAIL injection in a xenograft model using cervical cancer cells. These results suggest that USP8 may act as a tumor suppressor or as an oncogene depending on the cellular context.

## The role of USP8 in sperm acrosome formation

During spermatogenesis, post-meiotic spermatids undergo severe morphological changes leading to the formation of spermatozoa [101]. These include acrosome formation which is a key event that is tightly controlled. The acrosome is an acidic membrane-bound organelle of Golgi- and endosomal/lysosomal-derived origin containing substances that facilitate fertilization [102,103]. Intriguingly, during spermiogenesis USP8 re-localizes together with the sperm-specific heat shock protein 40 (HSP40)/DNAJ chaperone protein Msj-1 and proteasomes to the cytoplasmic surface of the developing acrosome maintaining this particular co-localization in mature spermatozoa [104,105]. Moreover, ESCRT-0/USP8/EEA1-positive vesicles were found to contribute to the development of the acrosomal vacuole suggesting that both the endocytic and the biosynthetic pathway are involved independently in acrosomogenesis resembling the biogenesis of lysosome-related organelles (LROs) [103,106,107]. In this context USP8 might directly link the developing acrosome to microtubules via its MIT domain. Moreover, the receptor tyrosine kinase MET was delivered as an USP8 target to the acrosome and finally to the post acrosomal segment (PAS) harboring sperm-borne factors involved in oocyte activation [106]. In accordance with these findings, mutations in the *USP8* gene might account for some cases of unexplained infertility in humans [108].

## Conclusions, outlook and therapeutic implications

Our overview of the current state of USP8 research emphasizes its versatile molecular, cellular and pathology associated functions. The characteristics of USP8 being a multidomain protein has allowed the attribution of functions according to relevant interaction partners such as STAM and CHMP proteins, and 14-3-3 molecules. Consequently, the regulatory role of USP8 in endosomal sorting of transmembrane receptors to MVBs has emerged as its canonical function. However, it is getting clearer, that USP8 most likely is involved not only in processes related to endosomal trafficking such as acrosome formation or autophagy, but also in mechanisms controlling unrelated functions such as DSB repair or DR-induced apoptosis. The analysis of USP8 function also remains to be expanded to other ESCRT-mediated events including viral budding, exosome formation or cytokinesis. Although USP8 expression is not limited to cytosolic fractions, but also found in the nucleus, only a few examples of its potential nuclear function have been reported. These include the interaction of USP8 with BRUCE in the regulation of DSB repair. The identification of nuclear USP8 targets has become particularly important in view of the high immunoreactivity of mutant USP8 in the nuclei of ACTH-secreting pituitary adenomas. However, the essential and multi-functional role of USP8 complicates the dissection of specific USP8-dependent cellular pathways as the manipulation of USP8 expression often evokes multiple effects which may be integrated in a rather pathway-unspecific readout. Thus, timing and degree of USP8 protein depletion have to be tightly controlled to provide optimal conditions for pathway-specific analyses.

With USP8 representing an essential protein degradation regulator which governs multiple pathways involved in cell cycle progression, apoptosis and genomic integrity its dysregulation may play a more important role in tumorigenesis and resistance to treatment than previously anticipated. Inhibition of USP8 also serves as a potential avenue to enhance proteasomal or autophagosomal degradation of aggregated proteins in neurodegenerative diseases [72]. Currently, two specific USP8 inhibitors have been identified: DUBs-IN-2 (IC50: 0.28 µM) which has been obtained by high-throughput screening followed by the generation of selective analogues [109], and Ubv.8.2CΔ2 (IC50: 4,8 nM) which has been engineered from combinatorial libraries of ubiquitin variants through optimization of the low-affinity interactions between ubiquitin and the enzyme [110]. Inhibitor treatment revealed that USP8 is required for growth of glioblastoma stem cells [111], multiple myeloma cells [112] and gefitinib-resistant non-small cell lung cancer cells [113] and demonstrated the existence of a therapeutic window in comparison with growth inhibition of control cells. Using mouse corticotroph tumor AtT20 cells DUBs-IN-2 also suppressed ACTH production and cell proliferation [114,115]. However, representing an essential gene, USP8 does not meet ideal requirements to serve as a druggable target and toxicity aspects will need to be tightly controlled. A more conductive avenue to target specific USP8-mediated pathways is the identification of pathway- or cell type-specific druggable downstream targets or effectors that modify USP8 function such as the kinase(s) and phosphatase(s) controlling phosphorylation of the 14-3-3BM in USP8. The observed limited proteolysis of USP8 as a consequence of *USP8* mutations in the exon encoding the 14-3-3 binding region uncovers an interesting regulatory mechanism to control its enzymatic activity. It will be interesting to see whether this is just a pathological phenomenon or the extreme of a cell-intrinsic regulation mechanism. As USP8 was shown to undergo limited proteolysis upon TCR stimulation it seems reasonable to suppose that its activity can also be regulated endogenously by phosphorylation triggered 14-3-3 binding. Further characterization of the USP8 paralogue USP50 [116], commonly regarded as a pseudo-DUB [6], may also prove valuable, as the perception of non-functional DUBS as important allosteric regulators and scaffolding proteins is just starting to emerge [117].

Taken together, USP8 represents a typical member of the multidomain USP family and uncovering its specific functions, regulatory principles and cell specific action might be exemplary for other members of the USP family.

**Perspectives**

- Importance of the field: USP8 represents a typical member of the multidomain USP deubiquitinating enzyme family with essential functions in protein trafficking and stability. Uncovering its specific functions, regulatory principles and cell-specific action might be exemplary for other members of the USP family and may lead to the identification of new avenues to target cancer or neurodegenerative disease.
- Summary of the current thinking: USP8 represents an essential DUB which governs multiple pathways involved in cell cycle progression, apoptosis and genomic integrity. The canonical role of USP8 is the regulation of endosomal sorting of transmembrane receptors via interaction with the ESCRT machinery. Mutations in *USP8* are associated with CD. CD is caused by ACTH-secreting pituitary adenomas leading to hypercortisolism. Stabilization of the EGFR has been identified as the underlying cause of CD triggered by activated mutant USP8. However, dysregulation of USP8 in tumorigenesis may not be limited to corticotroph adenomas and additional USP8-mediated mechanisms may contribute to tumorigenesis. Moreover, inhibition of USP8 may serve as a potential avenue to enhance proteasomal or autophagosomal degradation of aggregated proteins in neurodegenerative diseases.
- Future directions: The analysis of USP8 function remains to be expanded to other ESCRT-mediated events including viral budding, exosome formation or cytokinesis. Moreover, although USP8 expression is not limited to cytosolic fractions, but also found in the nucleus, only a few examples of its potential nuclear function have been reported. Representing an essential gene, USP8 does not meet ideal requirements to serve as a druggable target. A more promising avenue to target specific USP8-mediated pathways is the identification of pathway- or cell type-specific druggable downstream targets or effectors that modify USP8 function. Further insights into the mechanisms that regulate inhibitory phosphorylation of the 14-3-3BM in USP8 may uncover new modes to control USP8 activity in a specific manner.

## Abbreviations

14-3-3BM: 14-3-3 binding motif
ACTH: adrenocorticotropic hormone
ALIX: ALG-2 interacting protein X
AMSH: associated molecule with the SH3 domain of STAM
BRIT1: breast cancer susceptibility gene C terminus-repeat inhibitor of human telomerase repeat transcriptase expression 1
BRUCE: baculovirus IAP repeat (BIR)-containing ubiquitin-conjugating enzyme
CD: Cushing's disease
Chmp: charged multivesicular body protein
ci-M6PR: cation-independent mannose 6-phosphate receptor
DR: death receptor
DSB: double-strand break
DUB: deubiquitinating enzyme
EGFR: epidermal growth factor receptor
ESCRT: endosomal sorting complexes required for transport
ESCs: embryonic stem cells
GADS: GRB2 related adaptor protein downstream of Shc
GLI: glioma-associated oncogene
HH: Hedgehog
HIF1α: hypoxia-inducible factor 1-α
HRS: hepatocyte growth factor-regulated tyrosine kinase substrate
HSP: heat shock protein
KO: knockout
MIT: microtubule interacting and transport
MVB: multivesicular body
PTC: patched
RTK: receptor tyrosine kinase
SH3BM: SH3 binding motif
SMO: smoothened
STAM: signal transducing adapter molecule
SUMO: small ubiquitin-like modifier
TCR: T cell receptor
TRAIL: tumor necrosis factor-related apoptosis-inducing ligand
USP: ubiquitin-specific protease
VHL: von Hippel-Lindau
